# High-to-Low Spectral Mapping for Cross-System Feature Adaptation in Medical Hyperspectral Imaging

**DOI:** 10.3390/bioengineering13050549

**Published:** 2026-05-13

**Authors:** Javier Santana-Nunez, Max Verbers, Carlos Vega, Francesca Manni, Raquel Leon, Jesús Morera Molina, Juan F. Piñeiro, Alfonso Lagares, Luis Jimenez-Roldan, Gustavo M. Callico, Svitlana Zinger, Himar Fabelo

**Affiliations:** 1 Fundación Canaria Instituto de Investigación Sanitaria de Canarias (FIISC), 35012 Las Palmas de Gran Canaria, Spain; 2 Research Unit, Hospital Universitario de Gran Canaria Dr. Negrín, 35010 Las Palmas de Gran Canaria, Spain; 3 Institute for Applied Microelectronics (IUMA), Universidad de Las Palmas de Gran Canaria, 35001 Las Palmas de Gran Canaria, Spain (C.V.); (R.L.); gustavo@iuma.ulpgc.es (G.M.C.); 4 Instituto de Investigación Sanitaria de Canarias (IISC), 35012 Las Palmas de Gran Canaria, Spain; 5 Department of Electrical Engineering, Eindhoven University of Technology (TU/e), 5612 Eindhoven, The Netherlands (F.M.); (S.Z.); 6 Department of Neurosurgery, Hospital Universitario de Gran Canaria Dr. Negrín, 35010 Las Palmas de Gran Canaria, Spain (J.M.M.); (J.F.P.); 7 Department of Neurosurgery, Hospital Universitario 12 Octubre, 28041 Madrid, Spain (A.L.); (L.J.-R.); 8 Department of Surgery, Medicine Faculty, Universidad Complutense de Madrid, 28040 Madrid, Spain; 9 Instituto de Investigaciones Sanitarias (imas12), 28041 Madrid, Spain

**Keywords:** hyperspectral imaging, data mapping, feature adaptation, neurosurgery, brain cancer

## Abstract

Hyperspectral (HS) imaging has proven to be a promising intraoperative tool for tissue discrimination. However, obtaining representative datasets for intraoperative imaging remains challenging due to the complexity of surgical workflows and the sensitivity of the operating environments. Hence, developing new methods for cross-system feature adaptation could address this limitation. This work proposes a method for mapping high-resolution spectral data into lower-resolution sensor-conditioned domains, generating synthetic HS data that replicate the spectral features of the target system. We assessed the mapped data using public HS datasets and quantified spectral similarities using different metrics. Additionally, we evaluated the method with a HS classification framework for an intraoperative brain tumour classification problem. Results demonstrate that the synthetic data achieve high spectral alignment to original and actual data, captured with the target system. The brain tumour classification results show comparable performance between data modalities. Overall, this work provides a way to adapt existing HS datasets to complement newly acquired data, accelerating the development of artificial intelligence algorithms. This is particularly relevant in medical research, and especially in neurosurgery, where the complexity of acquisition environments limits the collection of large datasets.

## 1. Introduction

Brain and nervous system cancer was the 12th most common cause of cancer mortality worldwide in 2022, with 321,476 cases and 248,305 deaths reported globally [1]. The global mortality-to-incidence ratio ranks fifth highest among all cancer, at 77.24%, underlining the urgent need for improved treatment strategies. Alongside radio- and chemotherapy, neurosurgery is the standard treatment for malignant brain tumours. Maximising the extent of resection has been associated with reduced mortality rates and recurrence [2,3]. Recent evidence continues to support a positive association between greater extent of resection and improved survival in glioma patients, while highlighting the need to balance oncological benefit with the risk of neurological impairment [4]. Neurosurgery remains the primary treatment for brain tumour removal. The preoperative Magnetic Resonance (MR) images serve as the foundation for planning the surgical entry point. These MR images are also integrated with neuronavigation systems to guide the surgeon during tumour resection [5]. Additionally, the intraoperative visualisation is also increased using fluorescent agents such as 5-aminolevulimic acid (5-ALA) or fluorescein sodium, which help delineate tumour margins. To tailor the surgical approach, intraoperative pathological analysis is sometimes performed. A tumour biopsy is taken and sent to the pathologist, with results typically available within 30 min while resection continues. Other intraoperative tools have been used to improve guidance. These include intraoperative MR imaging [6,7], which introduces challenges related to patient safety and the need for special equipment, and intraoperative ultrasound [8,9], facing issues relating with 3D registration and misleading fluid artefacts. All these procedures aim to maximise tumour removal while preserving neurological function, ultimately improving postoperative patient outcomes.

Despite these advances, neurosurgeons face significant challenges given the nature of the tumour types and brain tissue. Gliomas, for instance, diffusely infiltrate surrounding tissue, making it hard for the specialist to distinguish tumour from healthy tissue—even with microscopic guidance [10] or fluorescent agents [11]. Furthermore, brain shift—caused by opening the skull, cerebrospinal fluid loss, tissue resection, and bleeding [12]—renders preoperative imaging less reliable during surgery. These limitations regarding physiological tissue as well as the current intraoperative guidance tools highlight the need for improved real-time intraoperative visualisation techniques to enhance surgical precision and patient outcomes.

In recent years, research on medical hyperspectral (HS) imaging (HSI) has seen significant advances [13,14,15]. This non-contact, non-ionising imaging modality captures a wide range of wavelengths, enabling the analysis of both spectral and spatial features. These capabilities allow for the exploitation of biochemical and structural differences between healthy and cancerous tissues.

The potential of HSI in neurosurgery has gained increasing attention [16,17,18], leading to new efforts in data acquisition and algorithm development. However, intraoperative data collection for brain tumour delineation remains challenging, particularly due to the need for high-quality, annotated datasets. Establishing a robust foundation for algorithm development would significantly accelerate progress in the field.

In medical HSI, three main acquisition strategies are used: spatial scanning, spectral scanning and snapshot imaging [15]. These approaches differ the order in which spatial and spectral information are collected, resulting in distinct advantages and limitations. Spatial scanning acquires all spectral channels in a single shot, moving the HS sensor spatially across the subject. It moves either at the pixel level with a whisk-broom camera, or at line level with a push-broom camera, providing the highest spectral resolution but being highly sensitive to motion and requiring high acquisition time. Spectral scanning instead captures full spatial frames at individual wavelengths, offering high spatial resolution while being prone to motion artefacts between spectral captures. Snapshot imaging, in contrast, captures the entire HS cube in a single exposure, enabling fast acquisition and reducing motion artefacts. This is at the expense of spatial detail within each spectral channel.

Recent trends in HSI for brain tumour research have focused on the development of real-time intraoperative diagnostic systems. The goal is to create a decision-support tool capable of delivering fast and accurate diagnostics, assisting neurosurgeons in making informed decisions, minimising errors, and reducing delays during surgery. The NEMESIS-3D-CM project [19] employed a HS snapshot camera, demonstrating, as a proof-of-concept, that informative data can be captured even with a reduced number of wavelengths. This enables neurosurgeons a real-time augmented reality visualisation tool at up to 21 frames per second. In contrast, the HELICoiD project [20] employed a HS push-broom camera, which captured data with high spectral resolution, providing more detailed spectral information but limited to single-cube captures.

However, both approaches have limitations. Snapshot cameras typically offer lower and fixed spectral and spatial resolution and involve complex optics. Push-broom cameras require moving optics for scanning, are susceptible to motion artefacts due to slower acquisition, and involve large hardware. Within the STRATUM project [21], Liquid Crystal Tuneable Filters (LCTFs) are chosen to overcome these limitations. LCTF systems offer greater spectral tuneability, contain no moving parts, and can be easily integrated into surgical microscopes, allowing HSI based on narrow-band illumination without positioning the main device directly over the patient. Additionally, they provide high spatial resolution and a tuneable number of bands, making them well suited for intraoperative imaging.

In this work, a novel method for mapping high-resolution spectral data into a lower-resolution sensor-conditioned domains is proposed to adapt existing HS datasets to complement newly acquired data. Particularly, the proposed method is validated in a specific use case by mapping data captured using the HELICoiD and ITHaCA acquisition system [20] (push-broom HS system) into the format used by the STRATUM acquisition system [21] (LCTF HS system).

This data mapping method aims to facilitate algorithm development, enhance classification robustness by generating a larger dataset, and accelerate progress in HSI research. Since the recovery of the exact underlying spectrum from a HS image is inherently non-unique, as different spectra can yield similar measured responses, the proposed method should not be interpreted as an exact physical reconstruction. Instead, it is intended as a cross-system transformation from spectrally dense to sparser HSI systems. Accordingly, the proposed framework does not aim to preserve or recover interpretable narrow-band tissue absorption features, but rather to generate task-consistent, sensor-conditioned domain data retaining discriminative features for classification purposes; therefore, it is suitable for artificial intelligence-based algorithm development using multiple datasets captured with different acquisition systems. The proposed method is first validated using a Zenith Polymer^®^ reflectance standard and a plastic dataset, allowing a comparison between synthetically generated LCTF data and actual data directly captured by the STRATUM system. This comparison will highlight the spectral differences and will help assess the fidelity of the transformation. Subsequently, previously acquired HS data of in vivo human brain tissue are mapped into the synthetic LCTF sensor-conditioned domain and evaluated using the commonly applied classification method for an intraoperative brain tumour classification problem. This two-step experiment plan (first through spectral comparison with known materials, and then through analysis of classification performance differences) provides a robust framework for assessing the cross-system feature adaptation approach, particularly for datasets where direct validation is not feasible. Ultimately, this work aims to support the research and development of real-time HSI algorithms for intraoperative decision-support tools in clinical environments by addressing one of the primary barriers to entry: limited HS data availability.

## 2. Materials and Methods

This section presents the HS data mapping methodology developed and the materials used for its validation. First, a theoretical description of the different phases of the method is provided. Then, the characteristics of the experimental setup employed to validate the proposed data mapping approach are described. Moreover, a set of HS datasets acquired with both source and target HS systems is introduced. These datasets, combined with a selection of metrics, enable the assessment of the feature adaptation accuracy achieved by the proposed method. Finally, a classification framework previously used for intraoperative brain tumour detection is proposed as an applicability test in a realistic clinical scenario. In this work, the term “synthetic HS data” refers to sensor-consistent measurements deterministically generated under known system response models and estimated irradiance.

### 2.1. Proposed HS Data Mapping Method

In this work, we propose a new method to map HS data acquired with one imaging system into synthetic HS data with the spectral characteristics of a different HS acquisition system. The objective is to use existing datasets with high spectral resolution to simulate the data that would be captured by a new HS system, allowing its performance to be evaluated and enabling algorithm development without requiring a large number of new acquisitions.

**Figure 1:**
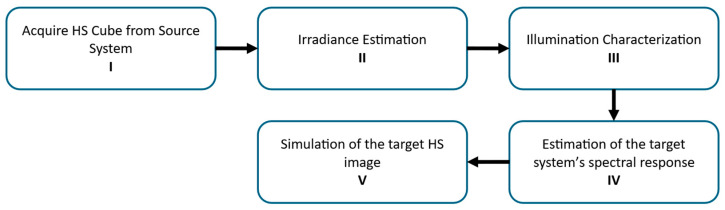
Block diagram of the proposed method for HS data mapping.

The proposed method for generating synthetic HS images for any target HS system from data captured with a source HS system with high spectral resolution consists of the following steps (Figure 1):

- Acquisition of the source HS data. Retrieve the raw HS data acquired with the source HS system, including all associated metadata.
- Irradiance estimation. Estimate the spectral irradiance reaching the source sensor by compensating for the quantum efficiency and optical characteristics of the source HS system.
- Illumination characterisation. Characterise the spectral distribution of the illumination used in the target HS system. In systems based on narrow-band illumination, the emitted light is measured independently for each spectral band to account for band-dependent illumination variations.
- Estimation of the target HS system’s spectral response. Model the effective spectral response of the target HS system by combining the measured illumination spectra (Step III) with the optical transmittance of the target system and the quantum efficiency of its sensor.
- Simulation of the target HS image. Generate the synthetic HS data by projecting the estimated spectral irradiance (Step II) through the target system’s spectral response (Step IV). This is performed independently for each wavelength defined by the target system and then they are combined into a single HS cube (synthetic HS image).

This procedure produces a raw synthetic HS image that approximates the data that would have been captured by the target HS system, under the assumptions of linear response and accurate spectral characterisation. Standard HS preprocessing steps, such as flat-field correction, remain necessary. For this reason, the procedure is applied to the raw source HS image and its corresponding dark and white reference images, after which the flat-field correction is applied to the synthetic data.

### 2.2. Experimental Setup

The proposed HS data mapping method has been experimentally validated using a high-spectral-resolution push-broom HS system as the source of the data to be simulated (the acquisition system employed in the HELICoiD and ITHaCA projects [20]), and a LCTF HS system with comparatively lower spectral resolution as the target of the simulation (the acquisition system developed in the STRATUM project [21]). Table 1 summarises the main characteristics of both HS systems. In the subsequent sections, each system is described in detail.

#### 2.2.1. LCTF HS Acquisition System

The LCTF HS acquisition system employed as the target reference for generating the synthetic LCTF HS data (and to capture actual data for spectral comparison) was developed in the context of the STRATUM project [22]. This system is able to acquire HS data by emitting filtered light using a customised LCTF illumination system, capturing spatial information using a monochromatic camera and performing a spectral scanning through different wavelengths to conform the HS image. Two different LCTF devices are used to cover the Visible and Near-Infrared (VNIR) range between 420 and 1100 nm: the Kurios K2VB1 (Thorlabs Inc., Newton, NJ, USA) for the Visible (VIS) spectral range (420–730 nm) and the Kurios K2XE2 (Thorlabs Inc., NJ, USA) for the Near-Infrared (NIR) spectral range (650–1100 nm). The use of both LCTFs allows the illumination system to filter light between 420 and 1100 nm with a minimum sampling interval of 1 nm, obtaining a maximum of 680 spectral bands. The LCTF devices are configurable regarding sampling interval and, hence, the obtained spectral bands.

The VIS LCTF has three user-selectable bandwidth settings of *Narrow*, *Medium*, and *Wide*. Table 2 shows the main differences between each of the three modes. The NIR LCTF has a fixed bandwidth of 13.26–24.11 nm. Since the Full Width at Half Maximum (FWHM) is wavelength-dependent ranging from 6 to 24 nm, and to avoid high redundancy without losing data on the final HS image, the sampling interval is set to 5 nm. This also helps to drastically reduce the acquisition time of the system, by also reducing the number of spectral bands to 137 (420–1100 nm).

**Figure 2:**
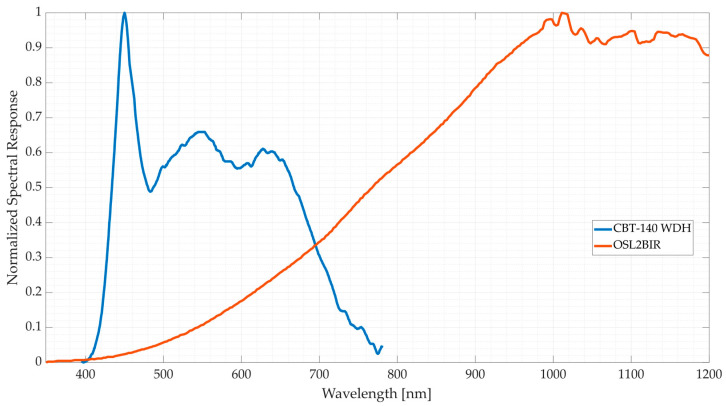
Normalised spectral response provided by the manufacturer of the CBT-140 WDH LED (blue) [25] compared to the OSL2BIR halogen lamp (orange) [26] in the VNIR range.

The use of two independent LCTFs allows enhancement of emitted light by selecting an optimised source of light for each LCTF’s range. While halogen lamps usually emit higher amounts of light on higher wavelengths (from ~600 to ~2200 nm), they are usually outperformed by high Colour Rendering Index (CRI) LEDs in the VIS range. Therefore, the CBT-140-WDH broadband white LED (Luminus Devices, Inc., Sunnyvale, CA, USA) and the OSL2BIR halogen lamp (Thorlabs Inc., NJ, USA) (Figure 2) were selected for the customised HS illumination system due to their high intensity response at the VIS and NIR ranges, respectively. Since both VIS and NIR LCTF devices have a sampling range overlapping between 650 and 730 nm, a middle transition band must be defined. Experimental results demonstrated that, within the overlapping region, the VIS LCTF provided higher transmitted light power at lower wavelengths. However, from 710 nm onward, the combined emitted light and system transmittance of the NIR LCTF exceeded the VIS LCTF device. Therefore, the transition band was established at 710 nm. This configuration allows an increase in the overall potential captured light, removing unnecessary redundancy and reducing the acquisition time.

This HS illumination system emits filtered light through a fibre optic light guide over a sample, and the reflected light is then captured by the camera sensor of the STRATUM system. The Kiralux LP126MU (Thorlabs Inc., NJ, USA) monochromatic camera is employed for this purpose due to its high spatial resolution (12.3 MP), providing 4096 × 3000 pixels per spectral band. Vega et al. [24] give a more extensive description of the LCTF system and show its performance against reference spectra and a snapshot HS system.

#### 2.2.2. Push-Broom HS Acquisition System

The push-broom HS acquisition system used as reference for acquiring HS data with high spectral resolution was developed in the context of the HELICoiD [27] and ITHaCA projects [28]. It is composed of a HS push-broom camera (Hyperspec^®^ VNIR A-Series, Headwall Photonics Inc., Fitchburg, MA, USA) with a Charge-Coupled Device (CCD) sensor (Adimec-1000 m, Teledyne Adimec, Eindhoven, The Netherlands). The system captures HS data within the VNIR range, covering from 400 to 1000 nm and acquiring 826 bands with an FWHM between 2 and 3 nm and a sampling interval of 0.73 nm. Spatially, this system is able to cover a maximum effective area of 129 × 260 mm with a spatial resolution of 741 × 1004 pixels, focusing on a working distance of 40 cm between the sample and camera lens. This results in a pixel size of 128.7 µm and a maximum acquisition time of 60 s. The illumination system employed is based on a Quartz–Tungsten–Halogen (QTH) lamp coupled to a cold light emitter. A more detailed description of this intraoperative HS acquisition system can be found in [29].

### 2.3. Method Implementation in the Experimental Setup

**Figure 3:**
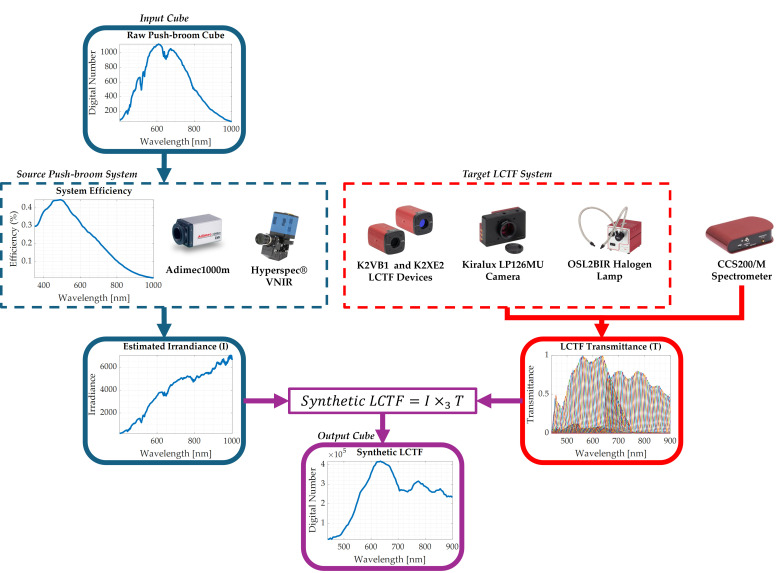
Application of the proposed method for generating the synthetic LCTF HS images from the push-broom HS data in the experimental setup.

For the application of the presented method in the proposed experimental setup (Figure 3), first, the spectral response and quantum efficiency of both acquisition systems are needed. For the push-broom HS camera sensor, the quantum efficiency curve was digitised from the manufacturer’s Operating and Technical Manual (revision 1.2) [30]. Owing to the narrow FWHM of the system’s spectral bands, the spectral response of each band was approximated as a Dirac delta function centred at its nominal wavelength.

The spectral response of the LCTF system for each band had to be physically measured to account for the efficiency of the optics and light sources. The emitted light at the output of the system was measured with a high-precision spectrometer (CCS200/M, Thorlabs Inc., NJ, USA), with a sampling interval of 0.25 nm with independent measurements for each band, represented as different curves in the LCTF Transmittance graph of Figure 3. A total of 280 captures were performed to obtain the light power density for each band and using the different configurations of the Kurios K2VB1 VIS LCTF device (*Narrow*, *Medium* and *Wide*). The resulting spectrometer measurements describe the spectral distribution of the light emitted by the LCTF system at each band, inherently including the influence of the illumination source.

The resulting spectra were arranged in matrix form, with dimensions corresponding to the number of wavelength samples and the number of spectral bands. Since the effective spectral range of the push-broom system is between 440 and 900 nm [31], instead of inspecting the selected 137 spectral bands, the transformation only covers 93 bands. Finally, the spectral response matrix was normalised using pixel-wise min-max [0-1] scaling.

The synthetic HS cube was obtained by first estimating the spectral irradiance (*SI*) of each pixel from the original raw push-broom HS pixel (*A*), compensating for the camera’s quantum efficiency (*QE*) as follows:

(1) SI=A/QE.

Then, the estimated irradiance was linearly interpolated to match the spectral resolution of the spectrometer. Due to the spectrometer’s denser spectral sampling compared to the source push-broom HS system, linear interpolation may introduce slight smoothing effects. However, these were found to have a negligible impact on data morphology.

Finally, the mode-3 tensor–matrix product ($\times_{3}$) [32] between the interpolated spectral irradiance (SI*) and the normalised LCTF spectral response (*SR*) matrix is determined according to:

(2) $$SP=SI^{*}\times_{3}SR,$$

generating a synthetic pixel (*SP*) in the LCTF sensor-conditioned domain, consistent with the spectral characteristics of the LCTF STRATUM HS system.

### 2.4. Hyperspectral Datasets

Three types of datasets were employed in this study to support the validation of the proposed HS data feature adaptation method: (i) *spectral characterisation dataset*, (ii) *plastic dataset*, and (iii) *in vivo human brain dataset*. Table 3 summarises the availability of each dataset type across the source push-broom HS system, the target LCTF system, and the synthetic LCTF data generated by the proposed method. As shown in the table, at this point there are no actual HS data captured using the LCTF system developed in the STRATUM project.

#### 2.4.1. Spectral Characterisation Dataset

**Figure 4:**
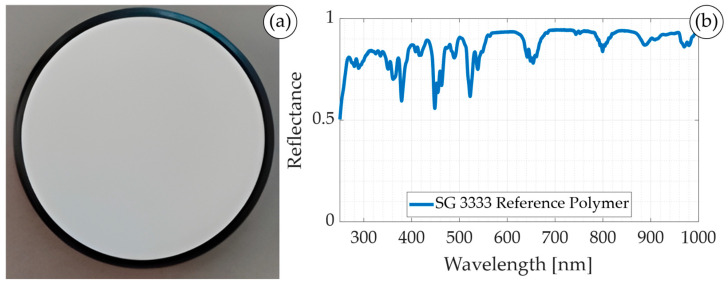
Spectral characterisation target. (**a**) Zenith Polymer^®^ SG 3333 and (**b**) its spectral signature.

A common approach to spectrally characterise any imaging system is to use a spectral characterisation target with known spectral signatures provided by the manufacturer. For this purpose, we employed the NIST (National Institute of Standards and Technology) traceable wavelength calibration standard, which is composed of a mixture of three pure, rare-earth oxides (holmium, erbium, and dysprosium) mixed into Zenith Polymer^®^ (LabSphere, Inc., North Sutton, NH, USA) [33]. This target (Figure 4a) provides well-differentiated peaks over the VIS and NIR ranges, previously validated using a spectrometer. The manufacturer provides the precise spectral response for each wavelength (Figure 4b), serving as reference to evaluate the accuracy of the synthetic LCTF HS data. In addition, the target was captured using both push-broom and LCTF systems.

#### 2.4.2. Plastic Dataset

**Figure 5:**
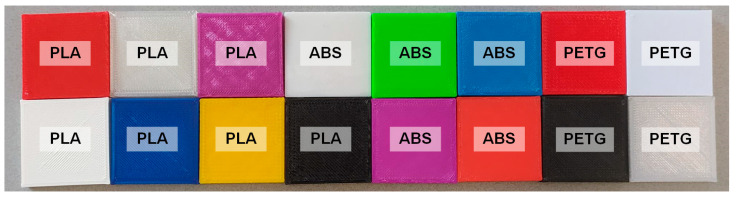
Sixteen square plastic samples of different colours and three materials: polylactic acid (PLA), acrylonitrile butadiene styrene (ABS), and polyethylene terephthalate glycol (PETG).

To validate the spectral response of the proposed methodology, a plastic dataset, previously acquired with the push-broom HS system [31], has been used. The dataset (Figure 5) is composed of sixteen different plastic cubes. Each sample has a unique combination of materials, polylactic acid (PLA), acrylonitrile butadiene styrene (ABS), or polyethylene terephthalate glycol (PETG), and different colours. This dataset was acquired using the LCTF system, enabling direct quantitative and qualitative comparison between push-broom HS data, synthetic LCTF data, and actual LCTF measurements in a variety of scenarios.

#### 2.4.3. In Vivo HS Human Brain Dataset

**Figure 6:**
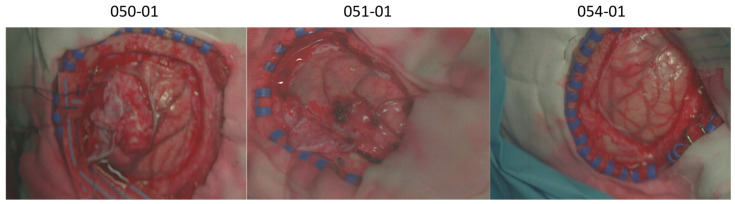
Examples of the synthetic RGB images from the in vivo HS human brain dataset.

The in vivo HS human brain dataset was employed in this study to evaluate whether the proposed HS data mapping method works and maintains the classification performance needed to distinguish between tumour and non-tumoral tissues using data captured intraoperatively during neurosurgical operations. The HS dataset (Figure 6) was acquired using the push-broom HS system in the framework of the HELICoiD and ITHaCA projects [20]. The dataset is composed of 61 HS images of exposed brain surface from 34 different patients. The HS images were acquired at the University Hospital of Gran Canaria Doctor Negrin, Spain. Written informed consent was obtained from all participants, and the study protocol and consent procedures were approved by the *Etica de la Investigacion/Comite de Etica de la Investigacion con Medicamentos (CEI/CEIM)* of the University Hospital Doctor Negrin (130069 and 2019-001-1). All research was performed in accordance with relevant guidelines/regulations. Given that actual LCTF HS images of in vivo human brain are not currently available, no direct comparisons can be performed. However, the push-broom HS data were processed using the proposed method to generate synthetic LCTF HS images and compare their classification performance with respect to the use of the original dataset.

### 2.5. Experimental Framework

**Figure 7:**
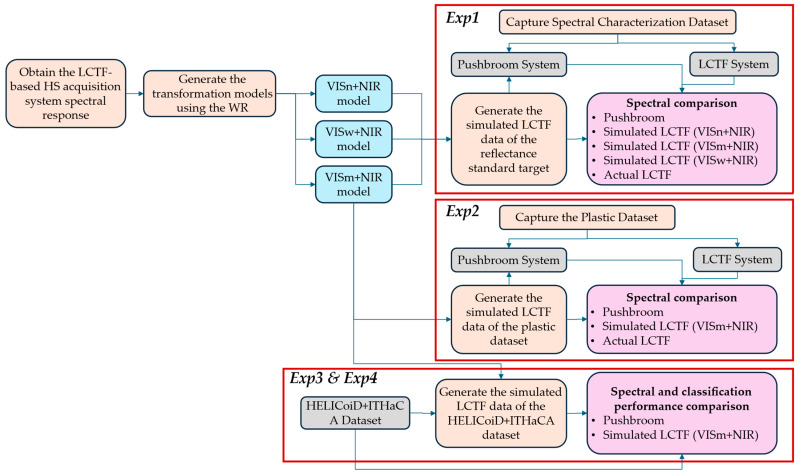
Proposed experimental framework to evaluate the proposed HS data mapping method. VISn: Visible LCTF configured in narrow-bandwidth mode. VISm: Visible LCTF configured in medium-bandwidth mode. VISw: Visible LCTF configured in wide-bandwidth mode.

The proposed HS data mapping method has been evaluated by performing a specific experimental framework shown in Figure 7. In this framework, the validation is structured along three complementary axes: (i) spectral consistency, (ii) system-specific behaviour, and (iii) task-level utility. Different experiments were carried out to evaluate the results using the previously mentioned datasets:

1. Experiment 1 (*Exp1*): Compare the spectral response of the synthetic LCTF HS data against the original HS push-broom data and the actual HS data captured by the LCTF acquisition system using the spectral characterisation dataset, shown in Section 2.4.1. In *Exp1*, three different transformation models are generated to evaluate the performance of the HS data mapping method when configuring the VIS LCTF device with the three user-selectable bandwidth settings (*Narrow*, *Medium* and *Wide*; see Section 2.2.1).
2. Experiment 2 (*Exp2*): Compare the spectral response of the synthetic LCTF HS data (using the *medium* bandwidth) against the original HS push-broom data and the actual HS data captured by the LCTF acquisition system using a plastic dataset composed of different materials and colours. These various scenarios would highlight any limitations of the mapping methodology.
3. Experiment 3 (*Exp3*): Compare the spectral response of the synthetic LCTF HS data (using the *medium* bandwidth) against the original HS push-broom data using an in vivo HS human brain database captured during neurosurgical operations.
4. Experiment 4 (*Exp4*): Evaluate the classification performance of the synthetic LCTF HS data (using the *medium* bandwidth) against the original HS push-broom data using a benchmarking processing framework to classify in vivo HS human brain images in four different classes (*normal*, *tumour*, *blood vessels* and *background*).

### 2.6. HSI Benchmark Classification Framework for Intraoperative Brain Tumour Detection

In previous works, the HELICoiD + ITHaCA dataset has been used to demonstrate, as a proof-of-concept, that intraoperative HSI is a promising decision-support tool for brain tumour identification and delineation during neurosurgical operations [20]. This framework employed the spectral and spatial information provided by HS data combining different algorithms for supervised classification, dimensionality reduction, spatial filtering and unsupervised segmentation.

**Figure 8:**
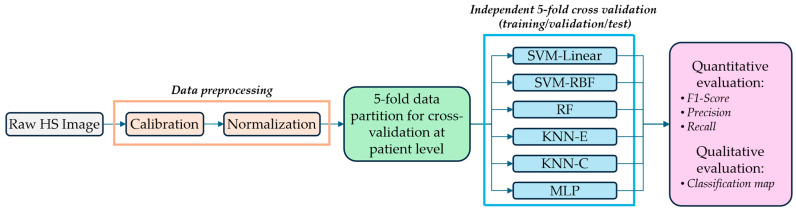
HSI benchmark classification framework for intraoperative brain tumour detection performance comparison. SVM: Support Vector Machines; RBF: Radial Basis Function; RF: Random Forest; KNN-E: K-Nearest Neighbours with Euclidean distance; KNN-C: K-Nearest Neighbours with Cosine distance; MLP: Multi-Layer Perceptron.

To test the capability of the target LCTF system to capture similar details without recruiting new patients, in this work, the supervised classification stage of such a framework is used as a benchmark to compare the classification performance when using the synthetic LCTF HS data instead of the original HS push-broom data (Figure 8). While many HSI classification algorithms exist, Leon et al. [20] used the original push-broom HS dataset employed in this work. Therefore, any statistical difference between both results would come as a result of the presented cross-system spectral mapping method.

In this framework, first, a calibrated HS image (*C_I_*) is obtained by preprocessing the raw HS image (*R_I_*) with the following equation:

(3) $$C_{I}=\frac{R_{I}-D_{I}}{W_{I}-D_{I}},$$

where a white reference image (*W_I_*) is captured from a white reference target that reflects the 95% of incident light, and a dark reference image (*D_I_*) is acquired by putting the cap on the camera lens to prevent light from entering the sensor. Next, the calibrated HS image is normalised by pixel to the minimum and maximum values of [0, 1]. All the preprocessed HS images are then partitioned into training, validation and test sets at the patient level, where each patient can contain more than one HS image, following a 5-fold cross-validation strategy as detailed in [20]. Using this data partition, four different supervised classifiers have been evaluated to measure the classification performance when using the synthetic LCTF images as input data for the intraoperative brain tumour detection task, as previously studied using the push-broom HS data in [20]. The following classifiers have been employed in this work:

• Support Vector Machines (SVMs): This classifier has the goal of separating the data classes by detecting the optimal hyperplane with a maximum margin that best separate the different classes. SVMs have been used in two different configurations, using the linear and Radial Basis Function (RBF) kernels (called SVM-Linear and SVM-RBF, respectively).
• Random Forest (RF): This decision tree classifier identifies the data classes by making a voting process using the predictions obtained from an aggregation of decision trees.
• K-Nearest Neighbours (KNN): This classifier compares each data sample with a certain number of neighbours (determined by a hyperparameter k) using a distance metric to find the nearest neighbour [34]. KNN has been used with the cosine and the Euclidean distance metrics (called KNN-E and KNN-C, respectively).
• Multi-Layer Perceptron (MLP): The MLP is a deep learning-based architecture which is composed of several layers, starting with an input layer, followed by one or more hidden layers and an output layer. Each layer conforms of a set of perception elements called neurons [35]. In our case, the architecture was designed with one hidden layer followed by a batch normalisation layer, using the rectified linear unit as an activation function.

The selected hyperparameters for each classifier are detailed in Table 4 and extracted from [20], and are applied in both push-broom and synthetic LCTF HS brain data. They have not been fine-tuned for each data modality to reduce sources of potential variability in the classification results.

The quantitative evaluation of the classification performance is based on the use of the ground truth of the test set using the evaluation metrics detailed in Section 2.7 (Equations (9)–(11)), comparing the results when using the push-broom HS data and the synthetic LCTF data. Qualitatively, the results are visually compared by generating the classification map of the input HS image (including both labelled and non-labelled pixels) with both data modalities.

These evaluations will expose any advantages or limitations of the target LCTF system to perform brain tumour identification without the recruitment of new patients. Previous works have pointed out that the reduction in spectral information would affect the classification accuracy [36]. Therefore, similar accuracy in classification performance would indicate that the synthetic HS data preserve task-relevant discriminative information, but not necessarily full spectral fidelity.

### 2.7. Evaluation Metrics

The evaluation metrics are divided into two categories: *mapping metrics* and *performance metrics*. The mapping metrics aim to quantify the similarity between pixels, whereas the performance metrics assess the performance of the push-broom and synthetic LCTF HS data classification. It is important to clarify the interpretation of these mapping metrics. For the datasets where acquisition of reliable ground-truth spectra is not feasible, these metrics are not interpreted as indicators of physiological spectral fidelity. Instead, they are exploited strictly as a pairwise consistency measure, quantifying agreement between push-broom, synthetic, and target-system measurements. Similar consistency-based spectral metrics have been widely used in HSI to assess measurement repeatability and cross-system agreement [37,38].

Mapping metrics are calculated to quantify differences between HS cubes, focussing on individual pixel spectra rather than spatial location. To achieve this, all pixels of a HS cube are flattened into an ordered matrix (*D*), which is as follows:

(4) $$D=\left(\begin{array}{cc} d_{i,j} & d_{i,w}\\ d_{p,j} & d_{p,w} \end{array}\right),$$

where $d_{i,j}$ represents the spectral value at pixel i and wavelength j, p is the total numbers of pixels, and w is the total number of wavelengths.

In this work, six mapping metrics have been computed at the pixel level: cross-correlation (5) computed on the 0th (original pixels), 1st, and 2nd spectral derivatives; Mean Square Error (MSE) per wavelength ($MSE_{w}$) (6) and per pixel ($MSE_{p}$) (7); and Spectral Angle Mapper (SAM) (8). These metrics are calculated as follows:

(5) $${Correlation}_{p}(i)=\frac{E\left\{\left(A_{i}-\mu_{A_{i}}\right)\left(B_{i}-\mu_{B_{i}}\right)\right\}}{\sigma_{A_{i}}\sigma_{B_{i}}},$$

(6) $$MSE_{w}(j)=\frac{1}{p}\sum_{i=1}^{p}{\left(a_{i,j}-b_{i,j}\right)}^{2},$$

(7) $$MSE_{p}(i)=\frac{1}{w}\sum_{j=1}^{w}{\left(a_{i,j}-b_{i,j}\right)}^{2},\;\mathrm{and}$$

(8) $$SAM_{p}(i)=\cos^{-1}\left(\frac{\sum_{j=1}^{w}a_{i,j}b_{i,j}}{\sqrt{\sum_{j=1}^{w}a_{i,j}^{2}}\sqrt{\sum_{j=1}^{w}b_{i,j}^{2}}}\right),$$

where *A* and *B* are the original push-broom and synthetic LCTF HS cubes, respectively. Furthermore, E denotes the expectation operator, which represents the average taken with respect to the joint distribution of the random variables. These metrics can be averaged to summarise overall error and highlight spectral ranges with poor mapping quality between both HS images.

Performance metrics focus on classification accuracy. Given the class imbalance in the brain dataset (e.g., few labelled tumour pixels), the Macro average is used. The F1-score is calculated as followed:

(9) $$F1=2\cdot \frac{Precision\cdot Recall}{Precision+Recall}\;,$$

where

(10) $$Precision=\frac{TP}{TP+FP},\;\mathrm{and}$$

(11) $$Recall=\frac{TP}{TP+FN}\;.$$

Here, TP, *FP*, and FN refer to true positive, false positive, and false negative, respectively. The classification accuracy between the different datasets is statistically evaluated using the paired Student’s t-test at 5% significance level and corrected for multiple comparisons using the Holm–Bonferroni method.

## 3. Experimental Results and Discussion

This section provides an overview of the performed experiments and their results. First, quantitative evaluations are performed to analyse the differences between push-broom, synthetic LCTF, and actual LCTF data. A range of evaluation metrics is used to assess variations and similarities in both spectral and spatial dimensions, as described previously as *Exp1*, *Exp2*, and *Exp3*. Second, classification is performed to quantitatively assess the performance of the push-broom and synthetic LCTF data using the HELICoiD and ITHaCA databases as described previously as *Exp4*. Together, these four experiments provide a comprehensive overview of the HS data mapping method and offer a thorough view of the feature adaptation results.

### 3.1. Quantitative Evaluation of the Spectral Response of the Synthetic LCTF HS Data

Three types of data are used for the quantitative evaluation of push-broom and synthetic LCTF data: (i) a spectral characterisation dataset, (ii) a plastic dataset, and (iii) the HELICoiD and ITHaCA dataset. The impact of the different operational modes (*Narrow*, *Medium*, and *Wide*) in the VIS LCTF device are analysed for the spectral characterisation (Zenith Polymer). Additionally, both spectral characterisation and plastic datasets are captured using the LCTF system in *Medium* mode, enabling a direct comparison between the actual LCTF HS data and their synthetic variants. In this section, all different data types are analysed separately to provide a detailed understanding of the feature adaptation.

#### 3.1.1. Spectral Characterisation Dataset Comparison (Exp1)

**Figure 9:**
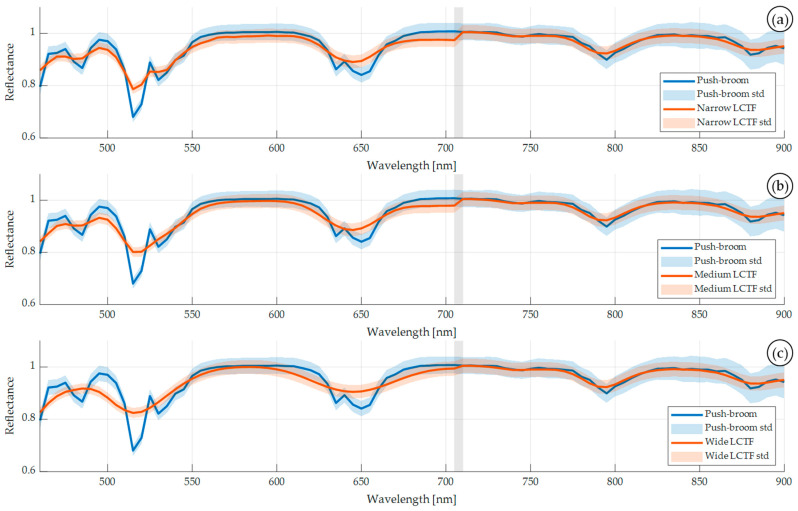
Original push-broom (blue) and synthetic LCTF (orange) average and standard deviation of the spectral signatures of the Zenith Polymer using the (**a**) *Narrow*, (**b**) *Medium*, and (**c**) *Wide* configurations. In grey is marked the LCTF transition range.

The three bandwidth modes for the VIS LCTF device (*Narrow*, *Medium*, and *Wide*) are evaluated using the Zenith Polymer and the previously described metrics (see Section 2.7). Figure 9 illustrates the mean spectral signature (solid line) and its standard deviation captured by the push-broom system compared with the synthetic LCTF data across the three bandwidth modes of the VIS LCTF device. Notable differences present themselves in the visible range of the synthetic LCTF HS data, i.e., from 440 to 710 nm, due to the bandwidth differences in this LCTF device. The FWHM of each wavelength is largest in the *Wide* configuration, resulting in a smoother and less specific spectral signature, with the most pronounced deviations occurring between 460 and 500 nm. Even in the *Narrow* configuration, a subtle smoothing effect is visible, indicating that the transformation systematically reduces the spectral specificity, which is expected.

Table 5 presents a quantitative comparison between the push-broom and synthetic LCTF HS data using the data within the 460–900 nm spectral range. This range was established according to previous works where the noise produced by the push-broom HS sensor was eliminated [39]. As expected, the *Wide* configuration led to greater information loss, particularly evident in the first and second order correlation metrics. These findings are consistent with the visual trends shown in Figure 9.

Overall, these results indicate that the mapping process yields minimal differences between push-broom and synthetic LCTF HS data, especially using the *Narrow* and *Medium* VIS LCTF configuration modes. Given that *Medium* mode requires lower acquisition time, as shown in Table 2, this is the optimal configuration for the STRATUM HS acquisition prototype, which has the goal of acquiring HS data during surgical procedures. Then, the *Medium* configuration will be used to capture the actual LCTF HS data of the Zenith Polymer and the plastic dataset and to for feature adaptation of the in vivo brain dataset.

While there is overall high performance, discontinuities appear at the wavelength region where the system transitions from the VIS LCTF to the NIR LCTF. These artefacts may reflect practical limitations of synthetic data mapping or a real feature of the actual LCTF HS system. Therefore, a comparison between synthetic and actual LCTF HS cubes is required. Unlike earlier experiments where pixel-wise comparisons were possible, next, to perform an evaluation of the synthetic against actual LCTF HS data captured with the target LCTF system, an average pixel spectrum per material to be compared is computed for both datasets.

**Figure 10:**
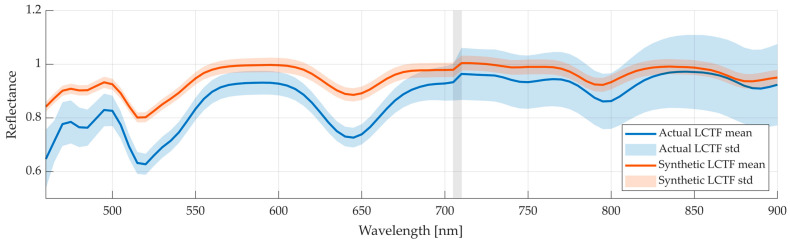
Actual LCTF captures (blue) and synthetic LCTF (orange) average and standard deviation of the spectral signatures of the Zenith Polymer using the *Medium* configuration. In grey is marked the LCTF transition range.

Figure 10 compares the spectra of data obtained from the actual LCTF system with those synthetically generated with the proposed method. Overall, the two spectra exhibit similar shapes, indicating that the transformation method preserves the general spectral features of the LCTF device. However, a noticeable offset is present, which is likely due to differences in illumination conditions or intensity calibration between setups. These illumination limitations in the actual LCTF system also result in a higher standard deviation in the NIR region (710–900 nm). Table 6 reports the quantitative similarity metrics for two comparisons, actual LCTF against synthetic LCTF, and synthetic LCTF against push-broom. While the $MSE_{p}$ is higher when comparing synthetic to actual LCTF measurements due to the presented offset, both correlation and SAM scores indicate a strong spectral similarity. Moreover, as shown in Figure 10, the spectral discontinuity found in the synthetic LCTF data also appears in the actual LCTF captures, indicating the high accuracy of the proposed mapping method and its capability to reproduce the spectral characteristics of the target HS system. The results highlight that the transformation preserves spectral shape reliably, which is important for tasks such as classification.

#### 3.1.2. Plastic Dataset Comparison (Exp2)

**Figure 11:**
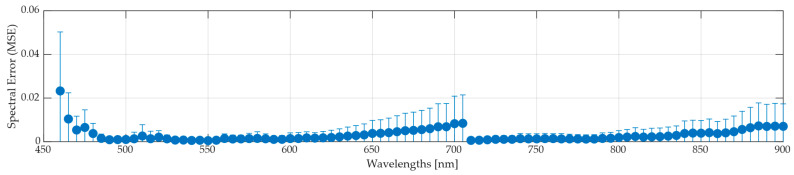
Average and standard deviation spectral $MSE_{w}$ results between push-broom and synthetic LCTF HS data of the entire plastic dataset using *Medium* bandwidth mode.

As in the Zenith Polymer analysis, the evaluation is conducted in two steps: First, comparing the push-broom data to the synthetic LCTF data; second, comparing the synthetic LCTF to the actual captures from the LCTF system. In this section, we focus on the *Medium* bandwidth mode, previously identified as the most suitable mapping configuration. To extend the interpretability of the Zenith Polymer analysis, an averaged spectral error is calculated to identify regions of elevated $MSE_{w}$ across the spectrum (Figure 11). Consistent with previous observations, higher errors are found at shorter wavelengths, particularly in the first spectral bands. Furthermore, the error increases after 800 nm, which aligns with the reduced signal-to-noise ratio in the original push-broom HS data at longer wavelengths. In contrast to previous analysis, the $MSE_{w}$ rises until the filter-switch wavelength and then decreases immediately after the transition. These behaviours show that the error progressively increases up to the filter-switch wavelength and drops to low error afterwards, showing that the boundary between filter regions affects the consistency of the spectral reconstruction.

Table 7 reports quantitative metrics, showing performance consistent with the Zenith Polymer for the $MSE_{p}$, SAM, and correlation metrics. Nevertheless, capturing finer spectral details proves more challenging, as reflected in the first- and second-order derivative correlation scores. For this larger and multi-image dataset, maintaining consistent spectral trends becomes increasingly difficult, leading to greater variation in these derivative metrics. The lower scores for first and second order indicate that subtle features, such as the rate of spectral change, are less accurately represented in the sensor-conditioned LCTF domain. Even so, other pixel-based error metrics remain relatively low, indicating that overall pixel fidelity is retained despite local spectral inconsistencies.

Table 8 presents the comparison between actual LCTF captures and synthetic counterparts, using an average spectra per colour and plastic type. Overall, coloured samples exhibit high correlation and low SAM score, indicating a strong similarity in spectral shape; green is a notable outlier in correlation. In contrast, pixel-wise $MSE_{p}$ scores are high, which is in line with illumination and calibration offsets increasing intensity-based errors while leaving shape-based metrics largely unaffected (Supplementary Materials Figure S1). This difference underscores that $MSE_{p}$ can be misleading when an offset is presented between the two spectra to be compared. The main cause of an offset is due to the illumination bias during the capture procedure. It should be noted that the correlation reflects similar spectral structure, whereas the SAM represents the spectral angle similarity without being affected by offset shifts. Plastics with near-flat spectra, i.e., black, white, and transparent, show poor correlation and $MSE_{p}$ score, yet relatively better SAM scores. Small noise or illumination fluctuations affect correlation for flat profiles heavily, while SAM remains more stable. An exception is the black plastics, which yield low $MSE_{p}$ due to reflectance values near zero. All reflectance of actual LCTF captures can be found in the Supplementary Materials Figures S2–S4.

**Figure 12:**
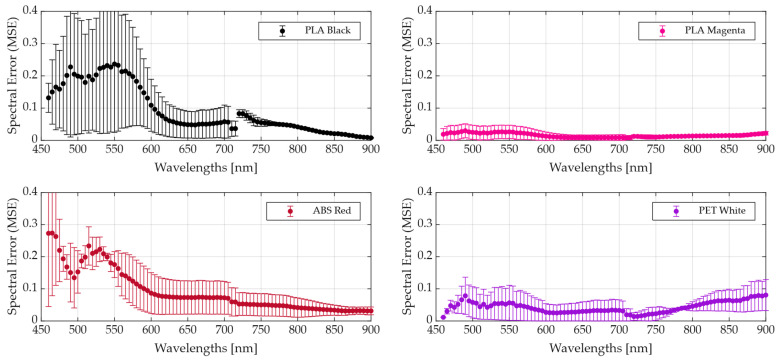
Average and standard deviation $MSE_{w}$ results between plastics of a synthetic LCTF and an actual LCTF HS image, both on an averaged pixel spectrum.

For further analysis, we examine spectral $MSE_{w}$ for four representative plastics in Figure 12. A full comparison between each pair of plastic types and colours can be found in Supplementary Materials Figure S5. The error shape suggests certain wavelengths are systematically harder to map; PLA Black and ABS Red (Figure 12, top-left and bottom-left, respectively) show the largest deviation in wavelengths lower than 600 nm. Notably, two spectral discontinuities appear around 710 and 720 nm, clearest in PLA Black, ABS Red and PET Red, which is consistent with the filter transition region.

#### 3.1.3. In Vivo Human Brain Dataset Comparison (Exp3)

**Figure 13:**
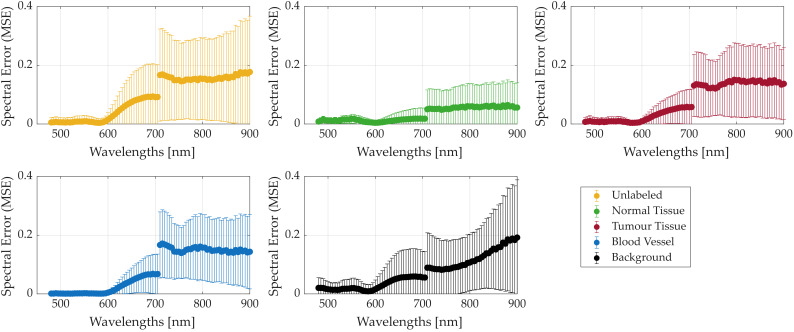
Average and standard deviation $MSE_{w}$ results per class between push-broom and synthetic LCTF HS data.

Similar to the analysis performed on the plastic dataset, Figure 13 presents the $MSE_{w}$ results for the brain dataset when comparing the push-broom HS data with respect to the synthetic LCTF HS data. In this figure, error values are shown per class, based on the ground truth labels, to better visualise which tissue types are more challenging to map. Unlike the other previous two experiments, the brain dataset shows larger discrepancies between push-broom and synthetic LCTF data in the NIR range, ranging from 710 to 900 nm. However, significant errors also persist in the VIS range, sometimes even exceeding those in the NIR region. It is worth noting that the VIS range commonly has lower intensities which can partially explain the lower error values in this range. Qualitative inspection of representative spectra in the sensor-conditioned LCTF domain indicates that major absorption trends are preserved, although no explicit band-wise quantitative analysis is performed.

Table 9 provides additional quantitative evaluation metrics. While all classes exhibit similar scores, minor differences distinguish some tissue types, indicating that the mapping method performs within expectations. Notably, correlation scores are high, suggesting that the spectral trends per pixel are well preserved between the push-broom and synthetic LCTF HS data.

**Figure 14:**
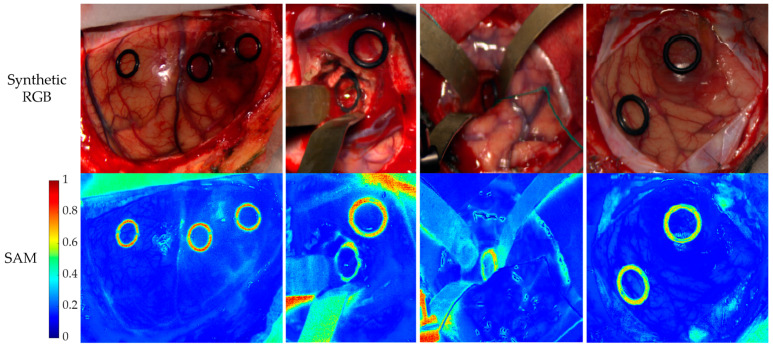
Synthetic RGB extracted from the HS images, emulating the human eye sensitivity (**top**), and SAM difference map per pixel between the original push-broom HS image and the synthetic LCTF HS image (**bottom**). The blue to red scale presents the SAM score, representing the spectral differences between the spectral signatures.

As a final step in the evaluation of the data mapping method for the brain dataset, an error map is constructed to spatially visualise pixel-wise differences. This provides qualitative insights into where feature adaptation discrepancies are most prominent. As an example, Figure 14 visualises these error maps for four different patients. Several visual features stand out as sources of error: *rubber rings*, *surgical metal tools*, *shaded areas*, *specular reflections*, *clean* or *bloodless cloths*, and *more blurry areas*.

### 3.2. Classification Performance Comparison Using the Synthetic LCTF HS Data (Exp4)

The feasibility and applicability of the proposed HS data mapping method in the in vivo HS human brain data were evaluated through a multiclass classification problem to differentiate between normal, tumour, blood vessels, and background pixels (see Section 2.7). Both push-broom HS data and synthetic LCTF HS data were calibrated, normalised using pixel-wise min-max [0-1] scaling, and classified using a 5-fold cross-validation approach developed in [20]. The hyperparameters, for both push-broom and synthetic LCTF HS datasets, of the multiple artificial intelligence models employed can be found in Table 4. The performance results obtained with these classifiers are summarised in Table 10.

Overall, the performance remains consistent between push-broom and synthetic LCTF HS data, with similar standard deviations across most metrics. While fold-level variation exists, the scores are comparable. The most notable difference occurs in the linear SVM, where the F1-score improves by 5% for synthetic LCTF data compared with push-broom data. To assess whether such differences were statistically significant, paired t-tests were conducted for each classifier and metric across five folds, followed by the Holm–Bonferroni correction to account for multiple comparisons. These tests confirmed that only the linear SVM exhibited a statistically significant difference between datasets (in F1-score and Tumour Recall, p<0.05). For all other classifiers, no statistically significant differences were observed. This suggests that the synthetic LCTF data preserve the discriminative spectral characteristics needed for reliable classification in this specific intraoperative brain tumour classification problem.

Across all methods, the synthetic LCTF HS data generally improve classification performance, likely due to the averaging effect of the data mapping method and the wider FWHM of the LCTF system, which acts as a Gaussian-like smoothing window and reduces spectral inconsistencies. Another observation is the high standard deviations for tumour precision, compared to recall (≈10%) and the precision of other classes (<10%). This variability suggests that classifiers struggle to maintain consistent tumour detection across folds, likely due to the limited dataset of 61 HS images. These findings reinforce the need for regularisation and data augmentation and improve data interoperability to build a larger, more robust dataset.

**Figure 15:**
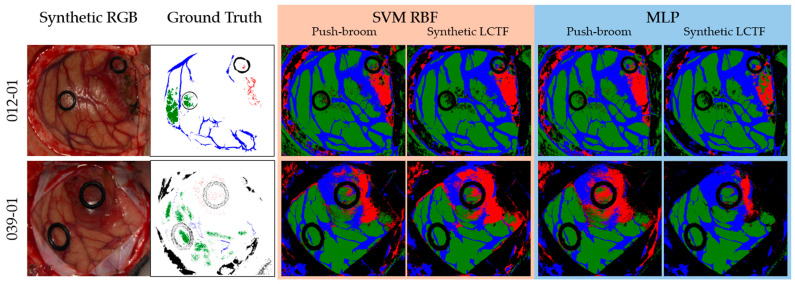
Example of two classification maps for patients 012-01 and 039-01 from the in vivo HS human brain dataset. Synthetic RGB and ground truth are shown together with the SVM RBF and MLP classification maps, of which the results are based both on the push-broom and synthetic LCTF HS data. The colours in the classification maps and ground truths are: green (normal tissue), red (tumour tissue), blue (blood vessels), and black (background).

Figure 15 presents the qualitative results (classification maps) of two different subjects generated from the push-broom and synthetic LCTF data using the SVM RBF and MLP classifiers since they provided the best results. The original ground-truths and the synthetic RGB images are also included for reference. Several differences between input data and classifiers are evident. For the tumour class, the SVM RBF applied to the synthetic LCTF data produces a larger tumour-labelled region compared to the push-broom data, whereas the MLP classifier results in fewer tumour-labelled pixels in the LCTF image. Additionally, the background class appears to be more spatially coherent in the LCTF-derived maps, showing fewer speckle-like misclassifications, which is most noticeable in the MLP classifier. This improvement is particularly visible in areas affected by specular reflections and in regions outside the parenchyma. In any case, these qualitative results can be only visually evaluated, since no complete ground-truth is available for each image of the brain dataset.

### 3.3. Discussion of the Results

Overall, the results are very promising: low $MSE_{w}$ scores indicate similarity at the individual wavelength level, high correlation coefficients demonstrate that the linear relationship is preserved, and low SAM scores confirm a close sensor-level spectral correspondence and alignment. Together, these findings show the robustness of the HS data feature adaptation method when applied to high-spectral-resolution push-broom HS data to obtain synthetic lower-resolution data consistent with an LCTF HS system. However, it should be noted that the comparison of the synthetic LCTF HS data and the actual LCTF measurements is based on averages of two spectra. As a consequence, the physical meaning and subtle intra-class differences caused by illumination, surface texture, acquisition noise or spatial heterogeneity are not captured in this mean spectrum.

Moreover, the synthetic LCTF spectra are generated under idealised pixel-specific assumptions, while the actual LCTF measurements are influenced by ambient illumination conditions and other external factors. These factors complicate the one-to-one comparison between simulated and real LCTF data and make it more difficult to accurately interpret at a fine pixel level.

The proposed HS data mapping method is widely applicable to different systems, although several considerations must be taken into account. In this work, the mapping reduces the original 826 spectral bands to 137, which is further trimmed to an effective range of 93 bands for the synthetic LCTF HS data. This reduction corresponds to a ratio of 6 to 1, meaning that sufficient spectral information is available to reconstruct the target band reliably. Expectedly, decreasing the spectral resolution of the original data, e.g., push-broom system, could degrade performance. Ratios below 2 to 1 [40] are likely to make reconstruction significantly more challenging and less informed.

While the extensive tests show that the proposed mapping method achieves the desired performance, this does not imply that the method always produces perfect results. Although system-specific differences are minimised as much as possible, real intraoperative environments introduce additional difficulties including uncontrolled ambient lighting, specular reflections, and variability inherent to surgical scenes. These factors can introduce discrepancies that are not fully accounted for by the mapping process.

## 4. Conclusions

The acquisition of a comprehensive and representative HS dataset during brain tumour surgery remains difficult due to the demanding workflow and the constraints of the intraoperative environment. This work contributes to the development of future HS-based clinical applications by introducing a novel high-to-low spectral mapping for cross-system feature adaptation, enhancing interoperability between HS datasets from different systems, and, hence, increasing dataset size and facilitating early validations. The introduced methodology uses the characteristics of the spectrally denser source HS system to estimate the illumination of the scene and combines it with the spectrally sparser target system’s features to obtain its synthetic response.

Across three datasets and four experiments, the results using a validation experimental setup demonstrate the robustness of the proposed mapping method. Pixel-wise spectral differences between push-broom and synthetic LCTF data remain minimal due to low MSE, high correlation, and low SAM scores. Furthermore, comparisons with real LCTF measurements show close agreement, mainly highlighted in the correlation coefficient and the SAM score. In the in vivo brain tumour HS data, which is the most relevant case, most deviations occur in non-tissue or blurry regions, confirming that the method behaves reliably in meaningful tissue area. The classification experiments reinforce these findings, showing comparable performance to the push-broom data, even reaching scores up to 5% better, using the same classification algorithms without a specific hyperparameter finetuning for the synthetic LCTF HS data. Therefore, the observed differences arise from the spectral characteristics of the LCTF system.

While the proposed methodology yields promising results, several limitations have been identified. First, the source HS system must provide higher spectral resolution than the target system in order to ensure sufficient spectral information for the transformation. As the spectral sampling of the source data becomes sparser, the precision of the synthetic spectra correspondingly decreases. Moreover, the proposed method does not take into account external factors such as illumination differences or acquisition inconsistencies. Therefore, the observed variability in the employed evaluation metrics should not be interpreted solely as a weakness of the presented method, but as the combined effect of model assumptions, target-system characteristics, and acquisition-condition mismatch. Although normalisation techniques could improve the performance of the evaluation metrics, they were not applied to maintain the interpretability of the reflectance and spectra within the limitations of the method.

Future works will explore the use of the proposed feature adaptation method for simulating the behaviour of different narrow-band imaging systems, including the same LCTF system operating in *Narrow* mode, enabling the emulation of responses at selected wavelengths. The resulting mapped data would support the assessment of this methodology for real-time retrieval of optical tissue properties (e.g., oxygen saturation maps, tissue haemoglobin index, and oxy- and deoxyhaemoglobin indexes) for intraoperative perfusion monitoring. Prior studies have demonstrated the relevance of these parameters for tissue characterisation across multiple medical applications [41,42,43].

A further direction involves biological validation through simultaneous acquisitions with both source and target hyperspectral systems on the same tissue samples. Such paired measurements are required to evaluate sensor-level consistency to overcome the limitations of indirect validation in the absence of real dual-system biological data.

Additionally, the synthetic LCTF HS dataset of in vivo human brain tissue will be leveraged to train artificial intelligence-based algorithms, subsequentially using them to classify actual LCTF HS data captured with the STRATUM acquisition system during neurosurgical procedures. This approach will increase the amount of data employed to generate the classification models and, hence, improve the generalisation of the models.

Overall, this work establishes a solid foundation for research on medical HSI. By enabling accurate high-to-low spectral mapping for cross-system feature adaptation from legacy acquisition systems, this method can expand available datasets and accelerate the development and validation of real-time HSI algorithms for intraoperative decision-support tools in clinical environments. This feasibility study lays the groundwork for future integration into surgical workflows, where rapid and reliable HSI analysis could assist in tissue classification, margin assessment, and pathology. Aligning data formats across systems is expected to accelerate algorithm development and enhance reproducibility and robustness, which are critical characteristics for clinical adoption.

## Figures and Tables

**Table 1:** Parameters of the use case acquisition systems for validating the proposed HS data mapping method.

Parameter	Push-Broom HS System	LCTF HS System
Spectral range (nm)	400–1000 (max)/440–900 (effective)	420–730 (VIS) + 650–1100 (NIR)/420–1100 (Total)
Sampling interval (nm)	0.73 (min)/3.61 (selected)	1 (min)/5 (selected)
FWHM (nm)	~2.5	6–13 (VIS)/13–24 (NIR)
Spatial resolution (pixel)	741 × 1004	4096 × 3000
#Spectral bands	826 (max)/128 (selected)	680 (max)/137 (selected)

**Table 2:** Key parameters of the Kurios K2VB1 user-selectable bandwidth settings [23].

Parameter	VIS LCTF Bandwidth Mode		
	Narrow	Medium	Wide
FWHM (nm)	7.1–14.4	10–26.9	14.6–49.8
Theoretical Peak Transmission (%)	1.01–27.6	1.45–31.43	1.69–36.24
Maximum Switching Speed (ms)	688.4	493.3	210.5

**Table 3:** Summary of the datasets and the HS system employed in the validation of the proposed method.

Dataset Name	Push-Broom HS System	Synthetic LCTF HS Data	LCTF HS System
Spectral Characterisation Dataset	x	x	x
Plastic Dataset	x	x	x
In Vivo Human Brain Dataset	x	x	

**Table 4:** Selected hyperparameters for each supervised classifier.

Classifier Name	Hyperparameter	Value
SVM-Linear	Cost	$2^{4}$
SVM-RBF	Cost	$2^{4}$
	Gamma	$2^{0}$
RF	Number of trees	50
KNN-E	Number of nearest neighbours	20
KNN-C	Number of nearest neighbours	20
MLP	Learning rate	0.01
	Number of neurons in hidden layer	100

**Table 5:** Average and standard deviation of the quantitative results obtained from the Zenith Polymer captured with the push-broom HS system against the synthetic LCTF HS data for the three bandwidth modes in the VIS LCTF device. Best values for different modes are highlighted in bold face. Comparisons made in the range 460 to 705 nm (VIS) and 710 to 900 nm (NIR).

Metric per Pixel	VIS LCTF			NIR LCTF
	Narrow	Medium	Wide	
$MSE_{P}$ (*1000)	**1.27 ± 0.179**	1.39 ± 0.190	2.12 ± 0.252	1.15 ± 0.324
SAM [rad]	**0.04 ± 0.003**	**0.04 ± 0.003**	0.05 ± 0.003	0.03 ± 0.050
Correlation	**0.93 ± 0.011**	0.90 ± 0.013	0.82 ± 0.020	0.65 ± 0.086
1st order correlation	**0.79 ± 0.030**	0.64 ± 0.034	0.44 ± 0.029	0.24 ± 0.076
2nd order correlation	**0.71 ± 0.049**	0.45 ± 0.048	0.23 ± 0.032	0.12 ± 0.074

**Table 6:** Quantitative results obtained from the Zenith Polymer captured with the actual LCTF system against the synthetic data and quantitative results obtained from push-broom against synthetic LCTF. Best values are highlighted in bold face. Comparisons made in the range of 460 to 900 nm.

Metric per Pixel	Actual LCTF and Synthetic LCTF	Push-Broom and Synthetic LCTF
$MSE_{P}$ (*1000)	8.510	**1.288 ± 0.175**
SAM [rad]	0.059	**0.037 ± 0.003**
Correlation	**0.948**	0.865 ± 0.020
1st order correlation	**0.966**	0.512 ± 0.039
2nd order correlation	**0.943**	0.305 ± 0.049

**Table 7:** Average and standard deviation of the quantitative results obtained from the plastic dataset captured with the push-broom HS system against the synthetic LCTF HS data for the *Medium* bandwidth mode.

Metric per Pixel	Scores
$MSE_{p}$ (*1000)	3.090 ± 2.010
SAM [rad]	0.056 ± 0.050
Correlation	0.931 ± 0.105
1 st order correlation	0.340 ± 0.207
2 nd order correlation	0.054 ± 0.142

**Table 8:** Average and standard deviation of the quantitative results obtained from the different coloured types of plastics in the dataset. Synthetic LCTF HS plastic data generated from push-broom data are compared against the actual HS data captured with the LCTF system. In bold are the best scores.

Colour	Plastic Type	Push-Broom Occurrences	Quantitative Metric		
			$MSE_{p}$	Correlation	SAM [rad]
Red	PLA	3	0.553 ± 0.328	0.908 ± 0.007	0.166 ± 0.051
	ABS	3	0.587 ± 0.072	0.942 ± 0.004	0.132 ± 0.004
	PET	3	0.329 ± 0.088	0.953 ± 0.012	0.177 ± 0.015
Green	ABS	1	0.338	0.765	0.246
Blue	PLA	2	0.322 ± 0.180	0.964 ± 0.004	0.198 ± 0.068
	ABS	2	0.351 ± 0.003	0.951 ± 0.002	0.255 ± 0.021
Yellow	PLA	1	0.477	0.931	0.104
Magenta	PLA	2	0.495 ± 0.105	0.982 ± 0.003	0.101 ± 0.000
	ABS	2	0.448 ± 0.044	**0.983 ± 0.000**	0.093 ± 0.004
Black	PLA	2	**0.022 ± 0.002**	−0.869 ± 0.012	0.317 ± 0.003
	PET	3	0.034 ± 0.031	−0.667 ± 0.097	0.232 ± 0.055
White	PLA	3	1.332 ± 0.273	−0.416 ± 0.210	0.092 ± 0.015
	ABS	4	0.570 ± 0.241	−0.044 ± 0.392	**0.061 ± 0.028**
	PET	2	1.077 ± 0.511	−0.676 ± 0.080	0.134 ± 0.019
Transparent	PLA	1	0.301	0.447	0.066
	PET	2	0.407 ± 0.010	−0.497 ± 0.085	0.094 ± 0.029

**Table 9:** Average and standard deviation of the quantitative results obtained from the brain dataset captured with the push-broom HS system against the synthetic LCTF HS data. NT: normal tissue; TT: tumour tissue; BV: blood vessels; BG: background; Unlabelled: every class not labelled in the original ground-truth; All: all classes (including unlabelled).

Metric	Pixel Class					
	All	Unlabelled	NT	TT	BV	BG
$MSE_{p}$	0.09 ± 0.077	0.09 ± 0.078	0.03 ± 0.037	0.07 ± 0.055	0.08 ± 0.054	0.07 ± 0.058
SAM [rad]	0.13 ± 0.085	0.13 ± 0.085	0.12 ± 0.042	0.11 ± 0.051	0.09 ± 0.029	0.16 ± 0.109
Correlation	0.98 ± 0.046	0.98 ± 0.046	0.99 ± 0.005	0.99 ± 0.007	0.99 ± 0.006	0.97 ± 0.060
1st order correlation	0.34 ± 0.170	0.34 ± 0.170	0.36 ± 0.122	0.34 ± 0.153	0.32 ± 0.108	0.41 ± 0.184
2nd order correlation	0.04 ± 0.126	0.04 ± 0.123	0.04 ± 0.134	0.04 ± 0.123	0.02 ± 0.114	0.06 ± 0.183

**Table 10:** Classification performance of the push-broom and synthetic LCTF HS data classification scores of the brain dataset. Best values are highlighted in bold font.

Classifier	HS Data Type	Macro F1	Tumour Precision	Tumour Recall
Linear SVM *	Push-broom	0.694 ± 0.021	0.503 ± 0.281	0.064 ± 0.039
	Synthetic LCTF	0.744 ± 0.015	0.524 ± 0.285	0.167 ± 0.051
RBF SVM	Push-broom	0.796 ± 0.034	0.477 ± 0.256	0.450 ± 0.132
	Synthetic LCTF	**0.803 ± 0.040**	0.490 ± 0.275	**0.477 ± 0.124**
RF	Push-broom	0.752 ± 0.030	0.420 ± 0.226	0.244 ± 0.120
	Synthetic LCTF	0.754 ± 0.025	0.426 ± 0.315	0.290 ± 0.087
KNN-E	Push-broom	0.747 ± 0.035	0.474 ± 0.262	0.255 ± 0.079
	Synthetic LCTF	0.750 ± 0.029	0.392 ± 0.267	0.323 ± 0.077
KNN-C	Push-broom	0.752 ± 0.041	0.435 ± 0.268	0.284 ± 0.099
	Synthetic LCTF	0.753 ± 0.032	0.398 ± 0.263	0.328 ± 0.067
MLP	Push-broom	0.790 ± 0.041	0.513 ± 0.305	0.357 ± 0.153
	Synthetic LCTF	0.776 ± 0.033	**0.530 ± 0.344**	0.316 ± 0.085

(*) Statistically significant difference (p<0.05) between the push-broom and synthetic LCTF HS data in Macro F1 and Tumour Recall metrics.

## Data Availability

The data presented in this study are available in the IUMA-ULPGC HSI Database and HSI Human Brain Database at https://hsidatabase.iuma.ulpgc.es/ (accessed on 1 March 2026) and https://hsibraindatabase.iuma.ulpgc.es/ (accessed on 1 March 2026). Additionally, the code and example data underlying this research can be found here: https://git.iuma.ulpgc.es:8300/stratum/public/high-to-low-spectral-mapping-for-cross-system-feature-adaptation/-/tree/5e29c790d2b410663d583eaf814e19f3e4faf7e3/ (accessed on 1 March 2026).

## Supplementary Materials

The following supporting information can be downloaded at https://www.mdpi.com/article/10.3390/bioengineering13050549/s1, Figure S1: Average reflectance and standard deviation for all available plastics. Actual LCTF measurements are shown in blue, while synthetic LCTF data generated from different push-broom captures are shown in orange, yellow, purple, and green. Figure S2: Average pixel reflectance of PLA plastic colours. Figure S3: Average pixel reflectance of ABS plastic colours. Figure S4: Average pixel reflectance of PETG plastic colours. Figure S5: Average and standard deviation for $MSE_{w}$ of all available plastics, calculated between synthetic LCTF and actual LCTF captures. No standard deviation available for PLA Yellow, PLA Transparent, and ABS Green due to having one comparison. Comparisons are made using the mean spectra per pair of plastic types and colours.
