# Supplementary material for: High-to-Low Spectral Mapping for Cross-System Feature Adaptation in Medical Hyperspectral Imaging
Source: Bioengineering (Basel). 2026 May 13;13(5):549. doi: 10.3390/bioengineering13050549 (PMC13203705; doi:10.3390/bioengineering13050549)
Supplement: Supplementary file 1 [file bioengineering-13-00549-s001.zip › bioengineering-4227715-supplementary.pdf]

Supplementary Information for:

High-to-Low Spectral Mapping for Cross-System Feature Adaptation in Medical Hyperspectral Imaging

Javier Santana-Nunez [1,2,3,4†,*], Max Verbers [5,†,*], Carlos Vega [3], Francesca Manni [5], Raquel Leon [3], Jesús Morera Molina [6], Juan F. Piñeiro [6], Alfonso Lagares [7], Luis Jimenez-Roldan [7], Gustavo M. Callico [3], Svitlana Zinger [5] and Himar Fabelo[1,2,3,4].

1 Fundación Canaria Instituto de Investigación Sanitaria de Canarias (FIISC), 35012 Las Palmas de Gran Canaria, Spain

2 Research Unit, Hospital Universitario de Gran Canaria Dr. Negrín, 35010 Las Palmas de Gran Canaria, Spain

3 Institute for Applied Microelectronics (IUMA), Universidad de Las Palmas de Gran Canaria, 35001 Las Palmas de Gran Canaria, Spain

4 Instituto de Investigación Sanitaria de Canarias (IISC), 35012 Las Palmas de Gran Canaria, Spain

5 Department of Electrical Engineering, Eindhoven University of Technology (TU/e), 5612 Eindhoven, The Netherlands

6 Department of Neurosurgery, Hospital Universitario de Gran Canaria Dr. Negrín, 35010 Las Palmas de Gran Canaria, Spain

7 Department of Neurosurgery, Hospital Universitario 12 Octubre, 28041 Madrid, Spain

8 Department of Surgery, Medicine Faculty, Universidad Complutense de Madrid, 28040 Madrid, Spain

9 Instituto de Investigaciones Sanitarias (imas12), 28041 Madrid, Spain

* Correspondence: jsnunez@iuma.ulpgc.es (J.S.-N.); m.verbers@tue.nl (M.V.)

† These authors contributed equally to this work.

Supplementary Figures

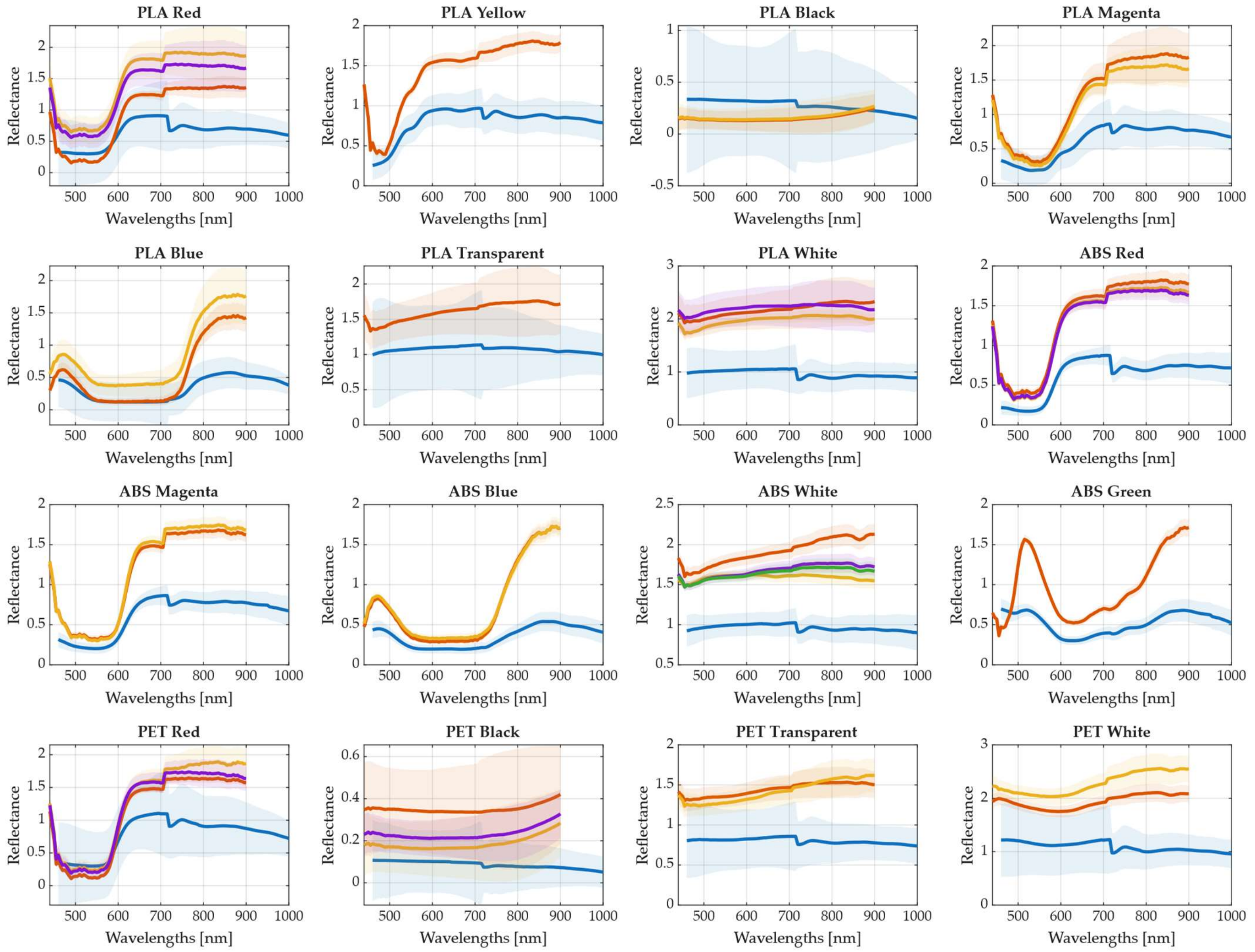

Figure S1. Average reflectance and standard deviation for all available plastics. Actual LCTF measurements are shown in blue, while synthetic LCTF data generated from different push-broom captures are shown in orange, yellow, purple, and green.

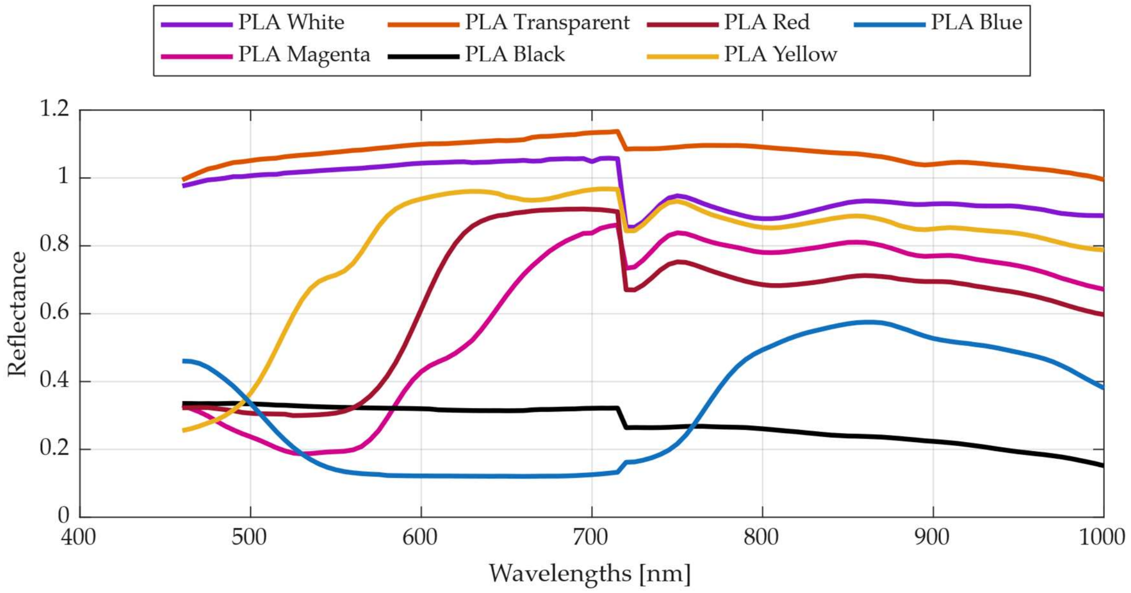

Figure S2. Average pixel reflectance of PLA plastic colours.

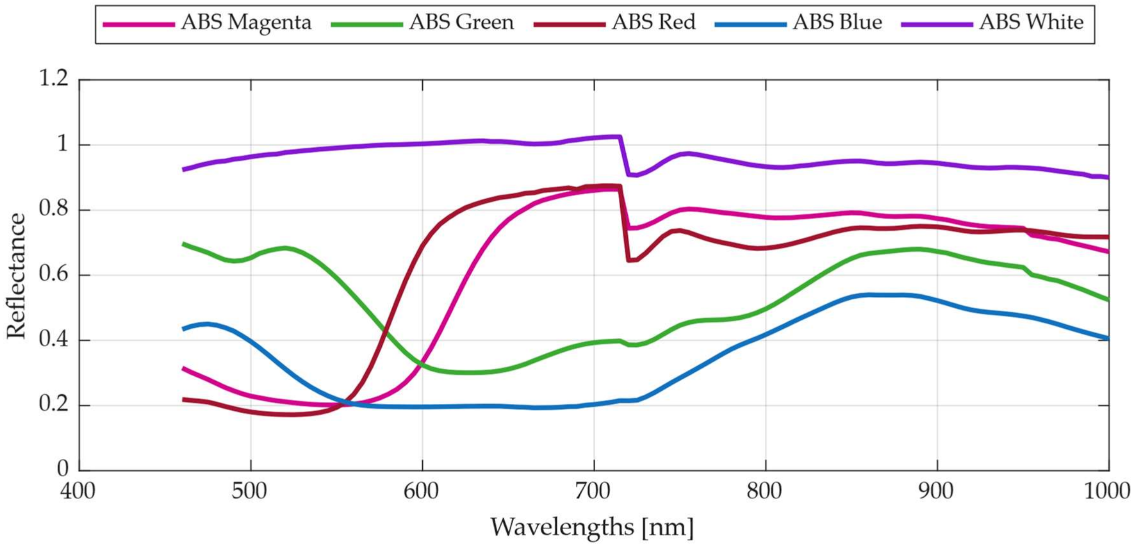

Figure S3. Average pixel reflectance of ABS plastic colours.

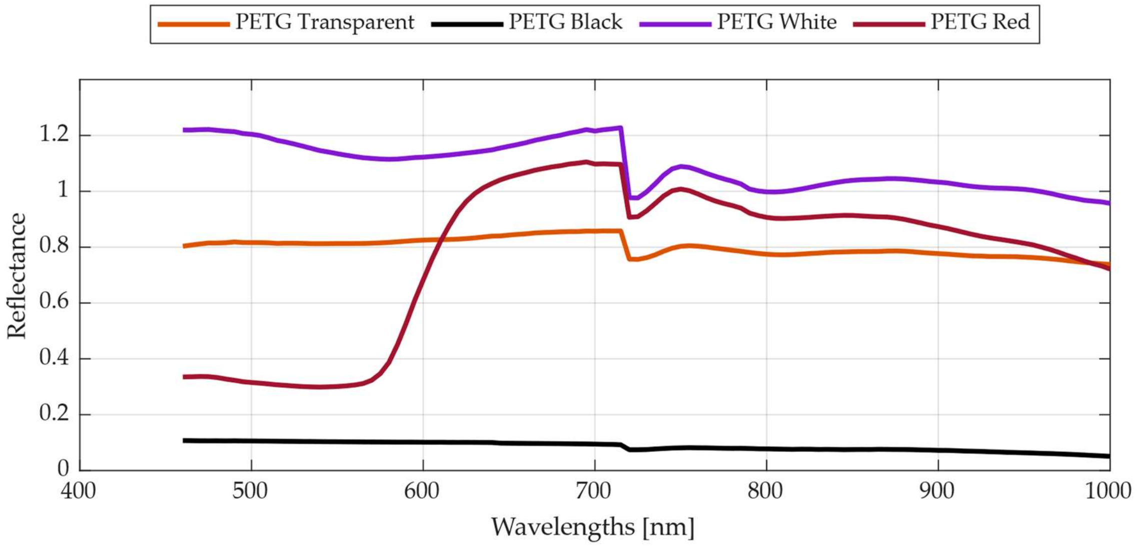

Figure S4. Average pixel reflectance of PETG plastic colours.

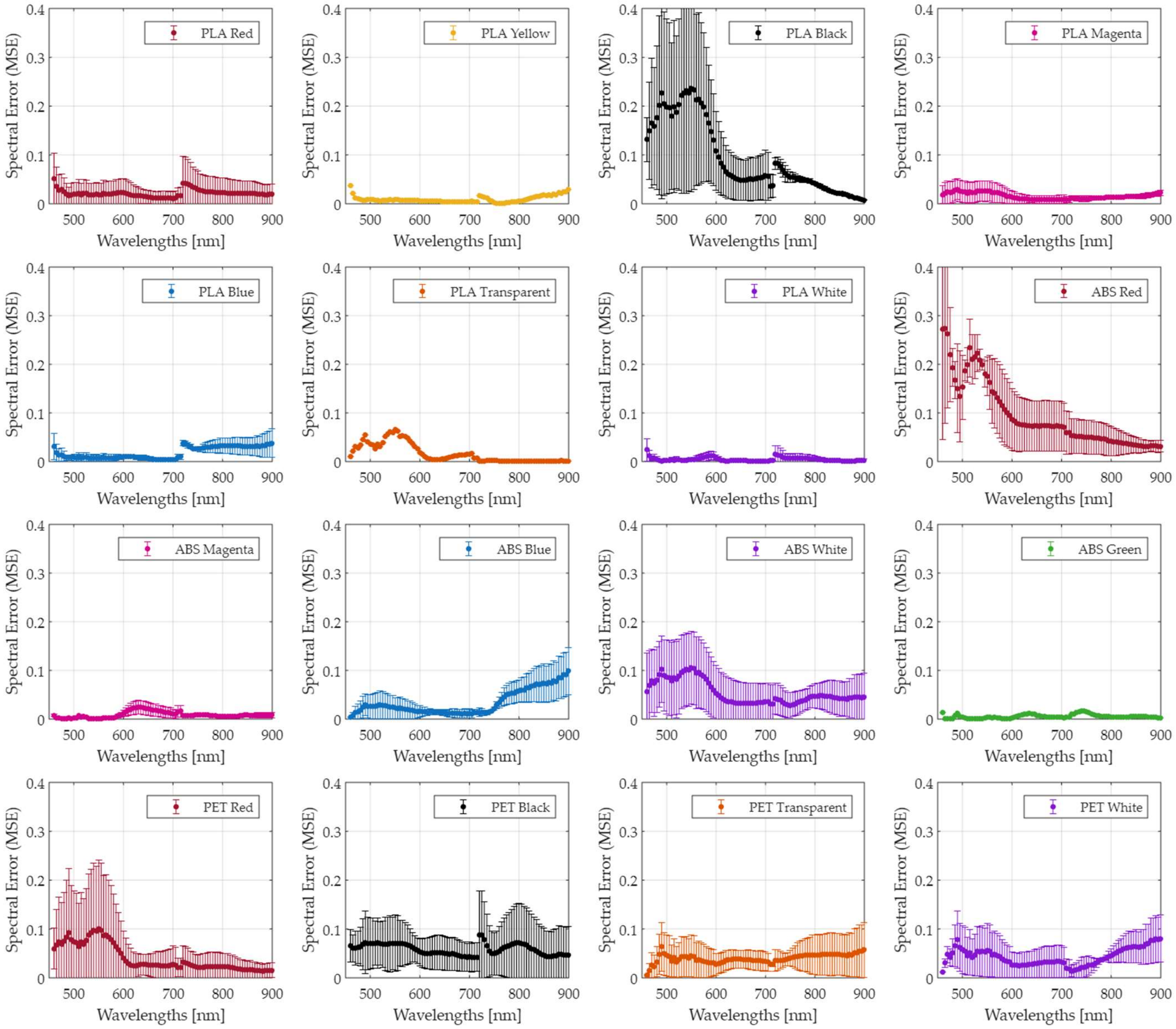

Figure S5. Average and standard deviation for MSE_w of all available plastics. Calculated between synthetic LCTF and actual LCTF captures. No standard deviation available for PLA Yellow, PLA Transparent, and ABS Green due to having one comparison. Comparisons are made using the mean spectra per pair of plastic type and colour.
